# Durable Impact of Computerized Clinical Decision Support for C. difficile Testing

**DOI:** 10.1017/ash.2025.322

**Published:** 2025-09-24

**Authors:** Stacy Park, Heather Cox, Lindsay Donohue, Amy Mathers

**Affiliations:** 1 University of Virginia Health System; 2 University of Virginia Health; 3 UVA Health

## Abstract

**Background:** Overdiagnosis of C. difficile in hospitalized patients is common and contributes to misdiagnosis, unnecessary treatment, and overestimation of nosocomial infection rates. Many institutions, including ours, have implemented computerized clinical decision support (CCDS) with reductions in testing rates, but long-term data on the impact of such interventions are limited. **Methods:** A previously reported CCDS intervention paired with education campaign and trainee financial incentive was implemented December 2016. A laxative alert was added in 2018 and testing changed from NAAT only to two-step testing in 2020. Hospital-onset C. difficile cases have been reviewed by members of the antimicrobial stewardship team in real time for diagnostic and antimicrobial prescribing opportunities for improvement (OFIs) since 2016, with a stable workflow for unit leadership notification and data entry in RedCap since June 2023. Diagnostic OFI categories are based on themes from early iterations of this case-based review process and include: clinical criteria not met, stool criteria not met, alternative explanation for diarrhea, smells like C. difficile, test of cure, duplicate test, delayed collection, delayed testing, and other. We analyzed reviews from 6/1/2023-12/31/2024 and further classified diagnostic OFI determinations as “No OFI”, “Inappropriate”, or “Appropriate with process OFI”. During the study period there was no ongoing financial incentive or concerted diagnostic stewardship educational campaign, though feedback continued to be provided to individuals and groups based on case reviews, and a single question regarding C. difficile testing was maintained in annual re-training. **Results:** There were 144 HO-CDI cases reviewed with no diagnostic OFI in 98 (68%). Testing was inappropriate in 16 (11%). Testing was appropriate with process OFIs in 30 (42%). The most common process OFIs were other-stool documentation (11), delayed testing (7), other-lack of discussion with preexisting ID consult (6), and delayed sample collection (5). In cases with delayed testing, earlier testing was not prevented by CCDS in any case. Conclusions: We found relatively low rates of inappropriate testing (11%) over a time period seven years out from initial implementation of CCDS without ongoing active house wide diagnostic stewardship initiatives. Carefully designed and implemented CCDS can be a valuable tool that facilitates sustained improvement and allows resources to be allocated to new efforts. We additionally observed no cases of delayed diagnosis attributable to CCDS with combination of established institutional criteria for testing and two-step testing.

